# Hospital Meetings

**Published:** 1905-03-25

**Authors:** 


### HOSPITAL MEETINGS.

### UNIVERSITY COLLEGE HOSPITAL.

The annual meeting was held on March 16th, his Grace the Duke of Bedford being in the chair. He gave a summary of the report for 1904 and some details of the high working cost of the new building, but this heavy expenditure, as the treasurer, Lord Monkswell, pointed out, would not seem so large when all was finished and the expenses spread over the whole working area. Unfortunately, there is a marked diminution in the subscriptions and donations, and legacies are not coming in as would be desirable, the consequence being that funded property has been sold out. In accordance with a wish of Lady Maple, a ward has been named the "Blundell Maple" ward, and her ladyship has endowed a bed with the sum of £1,000 in memory of her husband. Negotiations are still in progress to incorporate University College and the University of London. When these are completed and the Bill becomes law, the new corporation will be responsible for the management of the hospital and the school of advanced medical studies. To give effect to these new arrangements it was necessary to acquire new ground, and a site has been secured in Huntley Street, University Street, and Gower Street. Sir Donald Currie generously gave £100,000 to erect, on this site, the school of advanced medical studies, a nurses' home, and a house for students who attend to the maternity cases in the neighbourhood of the hospital. Still further buildings will shortly be begun for the accommodation of the obstetric staff, etc.

### ROYAL CHEST HOSPITAL, CITY ROAD.

Extensive Improvements Scheme.

The annual Court of Governors of the Royal Hospital for Diseases of the Chest, City Road, was held on the 15th inst., the Lord Mayor presiding. In moving the adoption of the report of the Council, together with the balance-sheet for 1904, he stated that the much-needed nurses' home, and the sanitary tower, for which a contract amounting to £6,987 had been entered into, were now in course of erection, and a very pleasing feature of the report was the announcement that, with a view to raising the necessary funds for this purpose, a gentleman, who for many years past had evinced a keen interest in the welfare of the institution, both as a member of the Council of management and as a generous supporter to the funds, had offered to give a donation of £2,000 towards the building fund, provided that an additional £3,000, which was necessary to meet the cost of the work, were subscribed by June 30th next. This generous donor, the Chairman added, was not the Lord Mayor of London, but he hoped that so munificent an offer would stimulate a vigorous effort on the part of all concerned, in order that it might be taken advantage of. An important improvement had been recently effected in the erection of balconies in front of the hospital, which afforded facilities for carrying out the open-air treatment. The chairman announced that he was about to place the Mansion House at the service of the hospital for an afternoon reception in May, when it was hoped some members of the royal family would be present.

Dr. W. H. White, in proposing a vote of thanks to the Council and various Standing Committees, said it was fitting that the proposal should be made by a member of the medical staff, since it gave him an opportunity of expressing the gratitude of the physicians and surgeons for the very generous way in which their suggestions were invariably received. As would be seen by reference to the report, the entire scheme of improvements suggested by the Hon. Medical Staff was an extensive one. It had, however, been formulated only after careful and prolonged deliberation, and the Medical Council were of opinion that the improvements were pressing, in order to enable the hospital to take its place among up-to-date institutions of the kind. They included, in addition to the building now in course of erection, improvements in the Out-patient Department, with the provision of adequate accommodation in waiting-rooms, etc., some enlargement of the dispensary, an isolation-room, a new operating theatre on the top floor, properly lighted, with anæsthetic, sterilising, and surgeons' rooms; improved quarters for the resident medical officers, domestic, and office staff; an *x*-ray room and apparatus, physicians' cloak-room, installation of electric light throughout the hospital, substitution of plain for stained glass, and new floorings to the wards. The architect's plans had been carefully considered, and the Medical Council did not hesitate to recommend them as an indication of the lines along which the improvement of the hospital should be undertaken as circumstances permitted.

### ROYAL DENTAL HOSPITAL.

At the annual meeting of the Royal Dental Hospital the chair was taken by Mr. John Tweedy, President of the Royal College of Surgeons, who gave an address on the aims and work of the hospital.

Mr. Tweedy glanced briefly at the figures shown in the report, all of which were satisfactory. A total of 99,458 cases had been treated during the year; of these, 42,000 had been allowed anæsthetics, which meant great lessening of suffering, but it also meant heavy increase of expense. It was gratifying to know that no casualties under anæsthetics had occurred in the institution for over 20 years. No one, said Mr. Tweedy, could over-estimate the importance of due attention to the teeth, especially with the young. It has been said that armies fight with their teeth, and statistics show that in 1903, 40 per cent. of recruits offering themselves for enlistment were refused on account of the defective state of their teeth. He further dwelt upon the noble and generous work done by the gentlemen who worked voluntarily in the hospital. Mr. Tweedy spoke of the regret every one felt at the resignation by Mr. Morton Smale who has held the office of Dean for 20 years.

The hospital has received from King Edward's Fund £250, from the Hospital Sunday Fund £213 14s., and the Hospital Saturday Fund £93 18s. 3d. Funds are needed to clear off what is called the chair debt; each chair with suitable adjustments costs £17 14s. 2d., and the public is asked to help in settling this debt, as well as to diminish the £1,000 due to the bankers and £700 still owed to the builders.

At the close of the meeting it was announced that Mr. Tweedy had himself given a cheque for £17 14s. 2d. to the Chair Fund.

Proceedings terminated with a vote of thanks to Mr. Morton Smale and Mr. Tweedy.

### THE FRENCH HOSPITAL.

The annual meeting of the above hospital was held on the 9th instant, the chair being taken by the president, M. E. Rüffer. After a short statement of the previous monthly meeting from the secretary, M. Pondepeyre, the report was read. The state of the funds seems on the whole satisfactory. The receipts for 1904 were £6,495 17s. 10d., being less than those of 1903 by £208 9s. 5d. This deficit, however, is accounted for by the fact that in 1903 a legacy of £800 was left to the hospital by the late M. Alexandre Haclin.

A long list of donations was given—among others that of the French doctors, who, at their recent visit to London, contributed in round figures £40.

An important addition has been made to the Convalescent Home at Brighton in the Pavilion Paul Cambon opened in last November. A new operating table in all respects up to date has been bought with the proceeds of a ball given by *L'Entente Cordiale*, which proceeds reached the sum of £32 14s. 4d.

Further freehold property has been acquired by the hospital consisting of three houses in Shaftesbury Avenue, and two in Great St. Andrew's Street, at a cost of £7,240 8s. 8d., the money being lent by Messrs. Rüffer to avoid the necessity of trenching upon the funded property of the hospital. M. Rüffer alluded feelingly to the great loss they had all sustained in the death of M. Achille Vintras who had laboured devotedly for the hospital for more than 40 years. A bust of this gentleman is in course of preparation, and it is hoped that subscriptions will be forthcoming to complete the memorial by a bronze cast.

### SAMARITAN FREE HOSPITAL FOR WOMEN.

The Rev. A. W. Oxford presided over the 58th annual meeting of the Samaritan Free Hospital for Women on the 14th inst.

By means of legacies amounting to £5,400, the loan of £1,000 borrowed in 1902 had been paid off, and £1,867 9s. 1d. had been added to capital account; the year, however, closed with an overdraft of £45 16s.; subscriptions had steadily diminished, and donations were considerably below the average, the usual appeal not having been sent to the public owing to the necessity for concentration of energy upon procuring funds for the new buildings. A large sum was still required for this purpose. Other improvements were also urgently needed, especially a theatre, an external fire-escape, and other alterations which would bring the hospital up to the requirements of the day. The committee earnestly appealed for at least £8,000 to enable them to undertake the most pressing of these improvements.

The report and accounts having been passed, the meeting closed with the usual votes of thanks.

A visit of inspection was then paid to the new building, situated behind the hospital, which consists of an outpatient department and sleeping accommodation for the nurses. On the ground floor there is a waiting-room 35 feet by 20 feet, and a consulting-room, out of which three white-tiled dressing-rooms open, these again communicating with the doctor's rooms. There is a glass roof, and ribbed glass has been used in the windows, with a view to admitting the maximum of light. The dispensaries are on the same floor, and are paved with terazzo. The two upper floors contain 16 bedrooms, with a bathroom on each floor, linen-room, cupboards, and outside fire-escape. Heating is effected by means of coils, and electric light is being installed. It is hoped that the new building may be finished by Easter.

### THE NEW HOSPITAL FOR WOMEN.

The annual meeting was held in the board room of the hospital. The chair was taken by Lady Frederick Cavendish, who proposed the adoption of the report and accounts for 1904. The motion was seconded by Colonel Bruce, R.A.M.C., F.R.S., who also pleaded eloquently the advisability of having a pathological laboratory attached to the hospital.

Lady Frederick Cavendish recalled her first visit to the hospital, which was then a modest affair in the Marylebone Road, as far back as the early seventies, when the appearance of women as physicians and surgeons caused wonder and surprise; since then, she said, the walls of the medical Jericho have indeed fallen before the energetic blasts of the weaker sex.

Mrs. Garrett-Anderson spoke on the responsibility of the committee in dealing with the contributions of the public, and the necessity of strict economy in every department, especially as the hospital was already in debt and struggling to overcome this undesirable state. The French doctors who visited London a short time since expressed their wonder to Mrs. Garrett-Anderson that English medical women took on the whole a better position than their French sisters, and the speaker replied that in her opinion it was greatly due to the fact that we had schools for the study of the profession apart from men, therefore the prejudice against medical women was somewhat less marked in our country.

The Rev. Prebendary Paget proposed the appointment of Lady Frances Balfour as Vice-President. He was seconded by Mrs. Scharlieb, M.D., and the motion was carried.

Mr. Paget, who is chaplain to the hospital and also a member of the committee, spoke in warm terms of the inner working of the institution, and deplored the coming loss of Miss Cox Davis, the matron, who goes to the Royal Free Hospital, just as a former matron, Miss Freeth, left to enter University College Hospital.

### ROYAL EYE HOSPITAL.

The annual meeting was held at the above hospital on the 15th inst., and the report and accounts of the past year were read and passed.

In the absence of Mr. W. Moresby-Chinnery, J.P., the President, the chair was taken by the Vice-President, Professor Malcolm M. McHardy, who gave some particulars of the working of the hospital. There are 40 beds and two cots, and a change that is thought very desirable is that children's ward should be built so that elderly and suffering pati nts should not be unnecessarily worried by the little ones' noise and boisterous spirits. Also further isolation accommodation is needed. Unfortunately, for the time it seems impossible that further extension can be made because ground cannot be purchased.

Proceedings terminated with the re-election of the council and the auditors and a vote of thanks to the medical and nursing staff, the treasurer, and the vice-president.

### ST. MONICA'S HOME HOSPITAL FOR SICK CHILDREN, BRONDESBURY PARK, N.W.

At the annual meeting of subscribers of this hospital (which was founded in the year 1874, for the reception of cases requiring a longer course of treatment than can be afforded in general hospitals) the chair was taken by the Rev. Francis Forster, M.A. The report stated that 128 patients had been under treatment during the year. The ordinary expenditure had amounted to £1,278, whereas the ordinary income was £1,106 only, including grants of £60 from King Edward's Hospital Fund, £67 from the Hospital Sunday Fund, and £35 from the Hospital Saturday Fund, which grants were specially valuable as showing that the work of the charity continued to meet with the approval of the councils of these important funds. The chairman made an urgent appeal for increased support to carry on the work of the charity efficiently.

